# Ablation treatment of hepatocellular carcinoma: a bibliometric analysis

**DOI:** 10.3389/fonc.2023.1166775

**Published:** 2023-06-22

**Authors:** Jianping Song, Tingxiao Zhang, Jianlei Wang, Yanfeng Liu

**Affiliations:** ^1^ Department of Hepatobiliary Surgery, The Second Hospital of Shandong University, Jinan, Shandong, China; ^2^ Department of Organ Transplantation, Qilu Hospital, Cheeloo College of Medicine, Shandong University, Jinan, Shandong, China; ^3^ Department of Hepatobiliary Surgery, Qilu Hospital, Cheeloo College of Medicine, Shandong University, Jinan, Shandong, China

**Keywords:** ablation, hepatocellular carcinoma, bibliometric analysis, Bibliometrix, Citespace, VOSviewer

## Abstract

**Objective:**

Ablation is a common treatment for hepatocellular carcinoma (HCC). This study aimed to assess research trends in the ablation treatment of HCC using bibliometric analysis.

**Methods:**

Publications between January 1, 1993 and December 31, 2022 were retrieved from the Web of Science database. The bibliometrix package from R software, CiteSpace, VOSviewer and an online analytical platform were used for data analysis and plotting.

**Results:**

A total of 4,029 publications were retrieved from the Web of Science database between 1993 and 2022. The annual growth rate of publication numbers was 10.14%. China had the largest number of publications in the field of HCC ablation. China and the United States of America have the most notable cooperation. Sun Yat-sen University had the largest number of publications in the field of HCC ablation. The most relevant journals were *Hepatology*, *Journal of Hepatology*, *Gastroenterology*, and *Radiology*. High-frequency keywords mainly focused on “therapy,” “resection,” “radiofrequency ablation” and “survival”.

**Conclusions:**

With the increase in related publications, the research direction of ablation treatment of HCC is mainly focused on “therapy,” “resection,” “radiofrequency ablation” and “survival”, and the ablation treatment method has gradually changed from percutaneous ethanol injection to radiofrequency ablation and microwave ablation. Irreversible electroporation may become the main method of ablation therapy in the future.

## 1 Introduction

HCC is the sixth most common malignant tumor and the fourth leading cause of cancer-related mortality worldwide (1). Orthotopic liver transplantation or radical resection is the best treatment option for HCC. However, owing to the insidious onset of HCC, it is often found in the late stage. Less than 20% of patients can undergo orthotopic liver transplantation or surgical resection, and most patients can only receive palliative treatment (2).

Ablation treatment can destroy tumors through different mechanisms: chemical method [percutaneous ethanol injection (PEI)], thermal method [radiofrequency ablation (RFA), microwave ablation (MWA), and cryoablation], and short pulses of high voltage (irreversible electroporation) and so on (3). Currently, the most commonly used ablation methods are RFA and MWA. Typically, ablation technology is considered a treatment method for HCC <2–3 cm in size (4). With technological progress and the promotion of systematic treatment of HCC, ablation not only has a therapeutic effect similar to that of surgical resection for small HCC but also is used in combination with other treatments for patients with unresectable advanced HCC (5). Ablation combined with TACE can significantly improve its therapeutic effect on certain HCC (6). Some studies have indicated that ablation combined with targeted therapy can significantly reduce the recurrence rate of early HCC (7, 8). Currently, studies have determined that ablation can enhance the treatment effect of immunotherapy in HCC (8, 9). Ablation has become an important part of the systematic treatment of HCC and has great development prospects

Bibliometric analysis is the process of analyzing the citation history of published papers, and it uses mathematical and statistical methods to quantitatively analyze a large number of studies in a specific research field, thereby revealing the aspects of and research trends in this field (10, 11). At present, many researchers use bibliometric analysis to evaluate their own research fields, which helps to quantify and to evaluate productivity in the field. Moreover, it can provide researchers with a comprehensive overview of the literature and identify potential research directions (12–15).

In recent decades, an increasing number of studies on ablation treatment of HCC have been published. However, to our knowledge, no bibliometric study on ablation treatment of HCC has been conducted. We did a bibliometric study to evaluate the literature on ablation treatment of HCC from January 1, 1993 to December 31, 2022 to describe the current state of the field and to identify new research directions.

## 2 Materials and methods

### 2.1 Search strategy

On January 3, 2023, we searched for relevant literature in the field of ablation treatment of HCC from January 1, 1993 to December 31, 2022 from the Core Collection database Web of Science (WOS) (data source: Science Citation Index Expanded). The main search terms were: (“Hepatocellular Carcinoma” OR “HCC” OR “Hepatoma” OR “Liver Cancer”) (Title) and (“ablation” OR “ablative” OR “RFA” OR “MWA” OR “Cryoablation” OR “Percutaneous ethanol injection” OR “PEI” OR “IRE” OR “irreversible electroporation” OR “HIFU” OR ‘high intensity focused ultrasound’) (Title). The search was restricted to English-language studies. The document types mainly included articles, reviews, editorial materials, proceedings, meeting abstracts, and letters. The articles which were early access or retracted were excluded. The main information regarding the publications is presented in **Table 1**.

**Table 1:** Main information of publications.

Description	Results
Timespan	1993:2022
Sources (Journals, Books, etc)	464
Documents	4029
Times Cited	83897
References	29405
Countries	57
Institutions	2216
Funds	1117
Authors	11919
Keywords	2493
Author’s Keywords	2899

### 2.2 Statistical analysis

All records and cited references from the WOS were analyzed using the bibliometrix package of R software (version 4.2.1), CiteSpace (version 6.1.6), VOSviewer (version 1.6.18) and an online analytical platform (https://bibliometric.com/ ). The biblimetrix package can convert and output simplified bibliographic information and complete data analysis and visualization (16). VOSviewer can generate the collaboration and co-citation networks. CiteSpace was used to analyze the burst keywords.

## 3 Results

### 3.1 Annual publication

**Figure 1:**
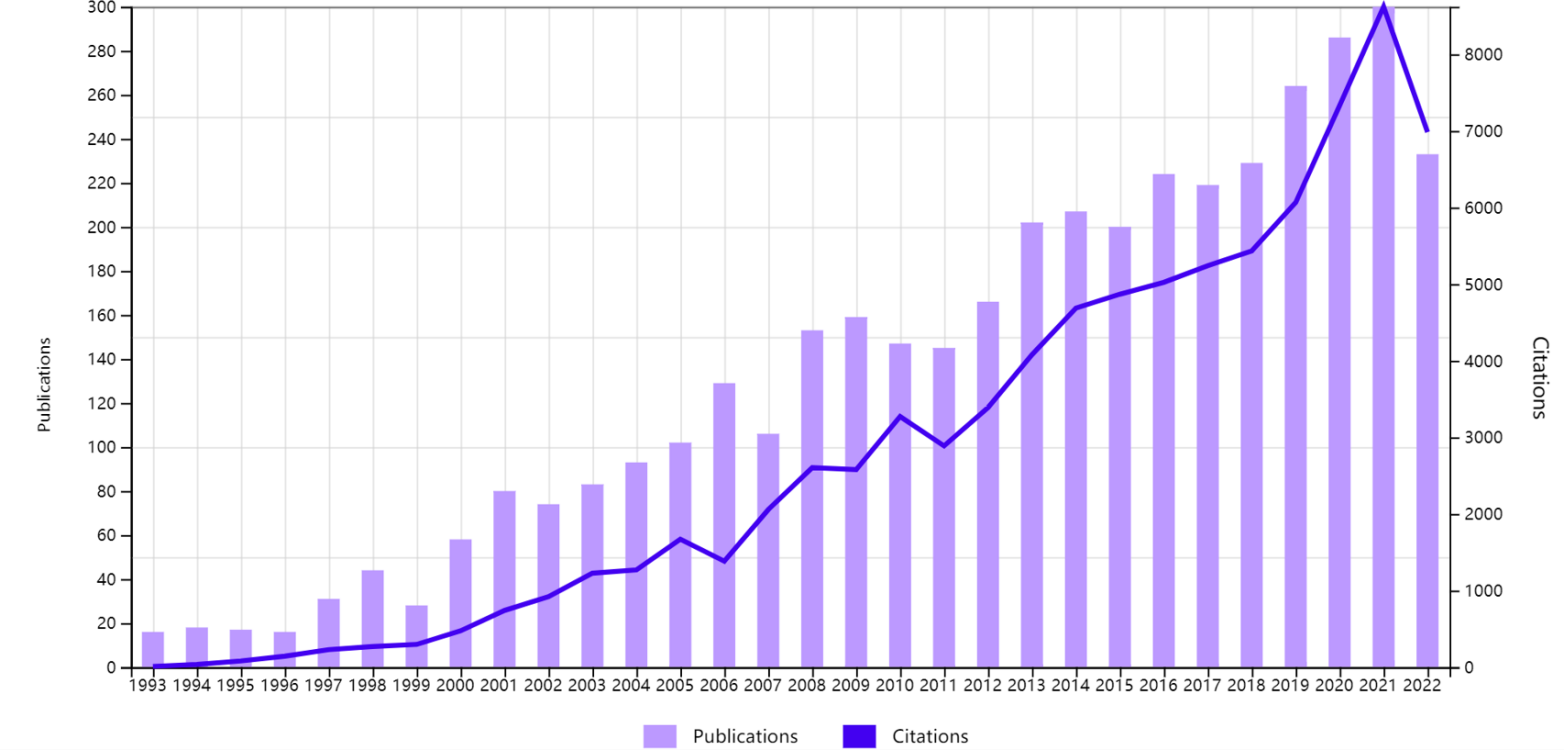
Trend of annual publication and citation numbers for ablation therapy of HCC from 1993 to 2022.

A total of 4,029 official publications from 1993 to 2022 were included, comprising 2,487 articles (61.73%), 51 proceedings (1.27%), 208 reviews (5.16%), 997 meeting abstracts (24.75%), 103 editorial materials (2.56%), and 183 letters (4.54%). Since 1993, the annual publishing volume has gradually increased, with an annual growth rate of 10.14% (**Figure 1**). Consistent with the current research trend in HCC ablation therapy, the annual cited frequency growth trend curve is also increased (**Figure 1**). Since the beginning of the 21st century, ablation therapy has been increasingly used in the treatment of HCC, and the number of articles is also increasing every year, demonstrating that ablation therapy of HCC has great development potential and clinical application prospects.

### 3.2 Countries/regions and institutions analysis

**Figure 2:**
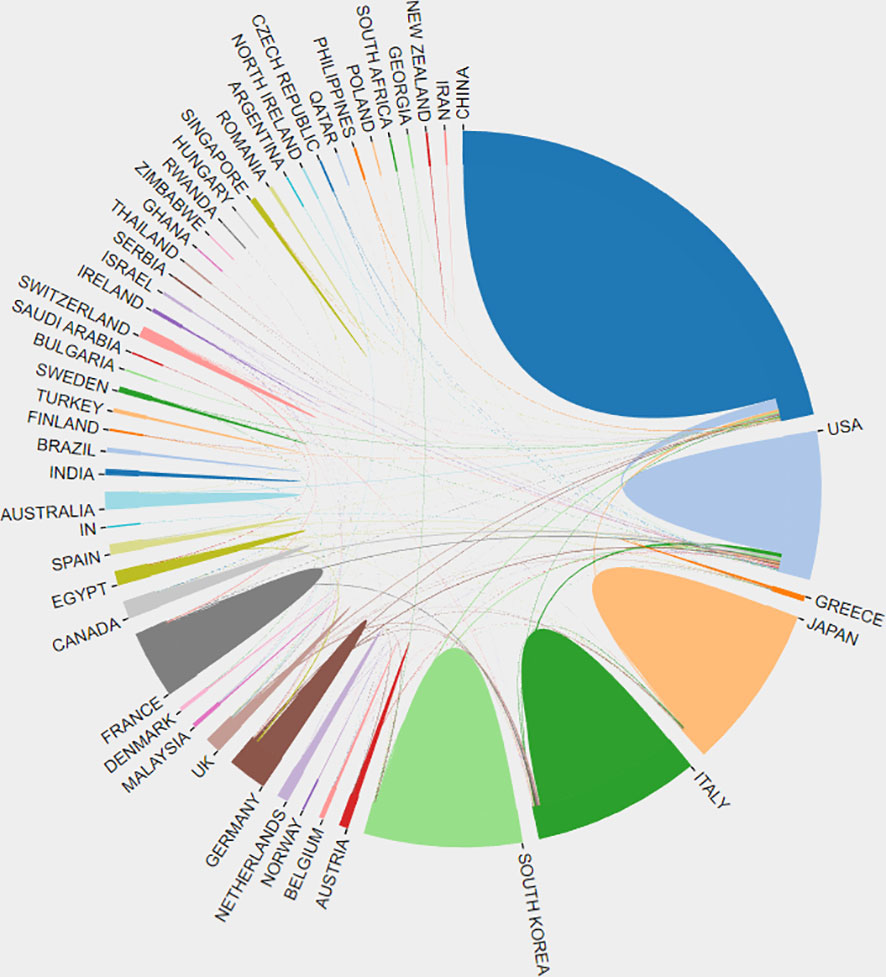
Countries/regions distribution and collaboration network.

From January 1, 1993 to December 31, 2022, 57 countries have contributed to HCC ablation research. The top 10 countries were China, Japan, Italy, the United States, Korea, France, Germany, the United Kingdom, Canada and Egypt (**Table 2**). Moreover, extensive cooperation between many countries/regions is also observed (**Figure 2**), most notably between China and the United States.

**Table 2:** Country production rank.

Rank	Country	Articles
1	China	1308
2	Japan	749
3	Italy	344
4	USA	333
5	Korea	326
6	France	80
7	Germany	78
8	United Kingdom	36
9	Canada	30
10	Egypt	29

**Figure 3:**
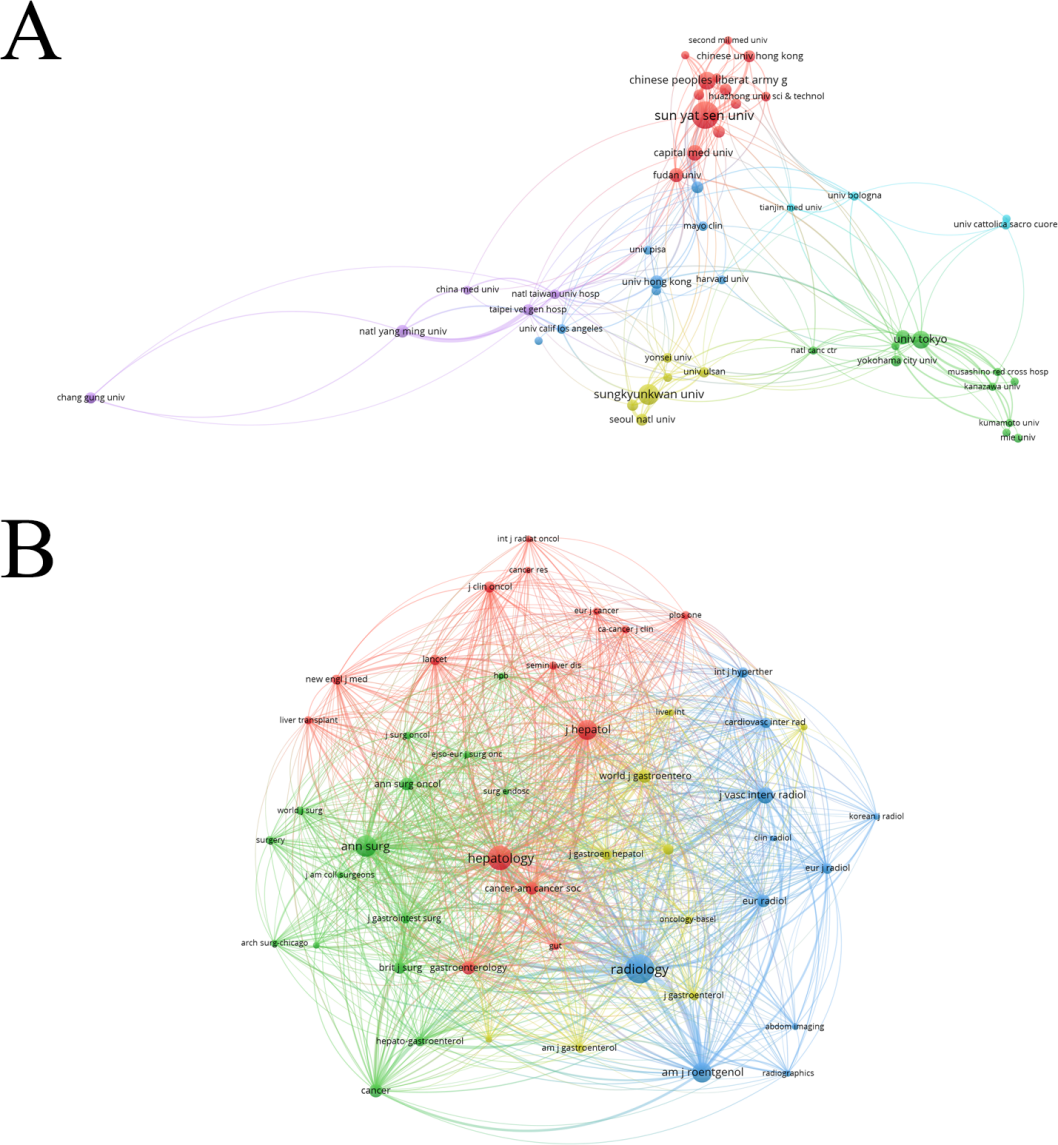
**(A)** Network visualization map of co-authorship institutions related to ablation treatment of hepatocellular carcinoma created by VOSviewer. **(B)** Network visualization map of co-cited journals related to ablation treatment of hepatocellular carcinoma created by VOSviewer.

The articles involved 2,216 institutions, and the top 10 institutions are Sun Yat-sen University, Sungkyunkwan University, Capital Medical University, University of Tokyo, General Hospital of the Chinese People’s Liberation Army, Fudan University, Seoul National University, University of Hong Kong, National Yang-Ming University, and Taipei Veterans General Hospital (**Table 3**). At present, the number of articles published by top two universities exceeds the total number of articles published by the other eight institutions. At present, institutions belonging to the same countries have close cooperation, whereas international exchanges and cooperation are relatively lacking (**Figure 3A**).

**Table 3:** Institution production rank.

Rank	Institution	Articles
1	Sun Yat-sen University	823
2	Sungkyunkwan University	487
3	Capital Medical University	190
4	University of Tokyo	188
5	General Hospital of the Chinese People’s Liberation Army	188
6	Fudan University	170
7	Seoul National University	169
8	University of Hong Kong	142
9	National Yang-Ming University	125
10	Taipei Veterans General Hospital	119

### 3.3 Relevant sources

Between January 1, 1993 and December 31, 2022, 464 journals published articles related to ablation treatment of HCC. Each of the top 10 most relevant journals published at least 72 articles. The most represented journals were *Hepatology*, *Journal of Hepatology*, *Gastroenterology*, and *Radiology* with publication numbers of 269, 196, 177, and 139, respectively (**Table 4**). At present, the most influential journal in the field of HCC ablation therapy is *Radiology*, with an H-index of 49. Other influential journals include the *American Journal of Roentgenology* and *Journal of Hepatology* (**Table 4**). There is a close citation relationship between these journals (**Figure 3B**).

**Table 4:** Journals production rank.

Rank	Journals	Articles	H-index
1	*Hepatology*	269	27
2	*Journal of Hepatology*	196	30
3	*Gastroenterology*	177	7
4	*Radiology*	139	49
5	*International Journal of Hyperthermia*	126	20
6	*Journal of Gastroenterology and Hepatology*	114	23
7	*Journal of Vascular and Interventional Radiology*	91	27
8	*American Journal of Roentgenology*	89	37
9	*Hepato-Gastroenterology*	78	19
10	*Journal of Clinical Oncology*	72	9

### 3.4 Citation analysis

**Figure 4:**
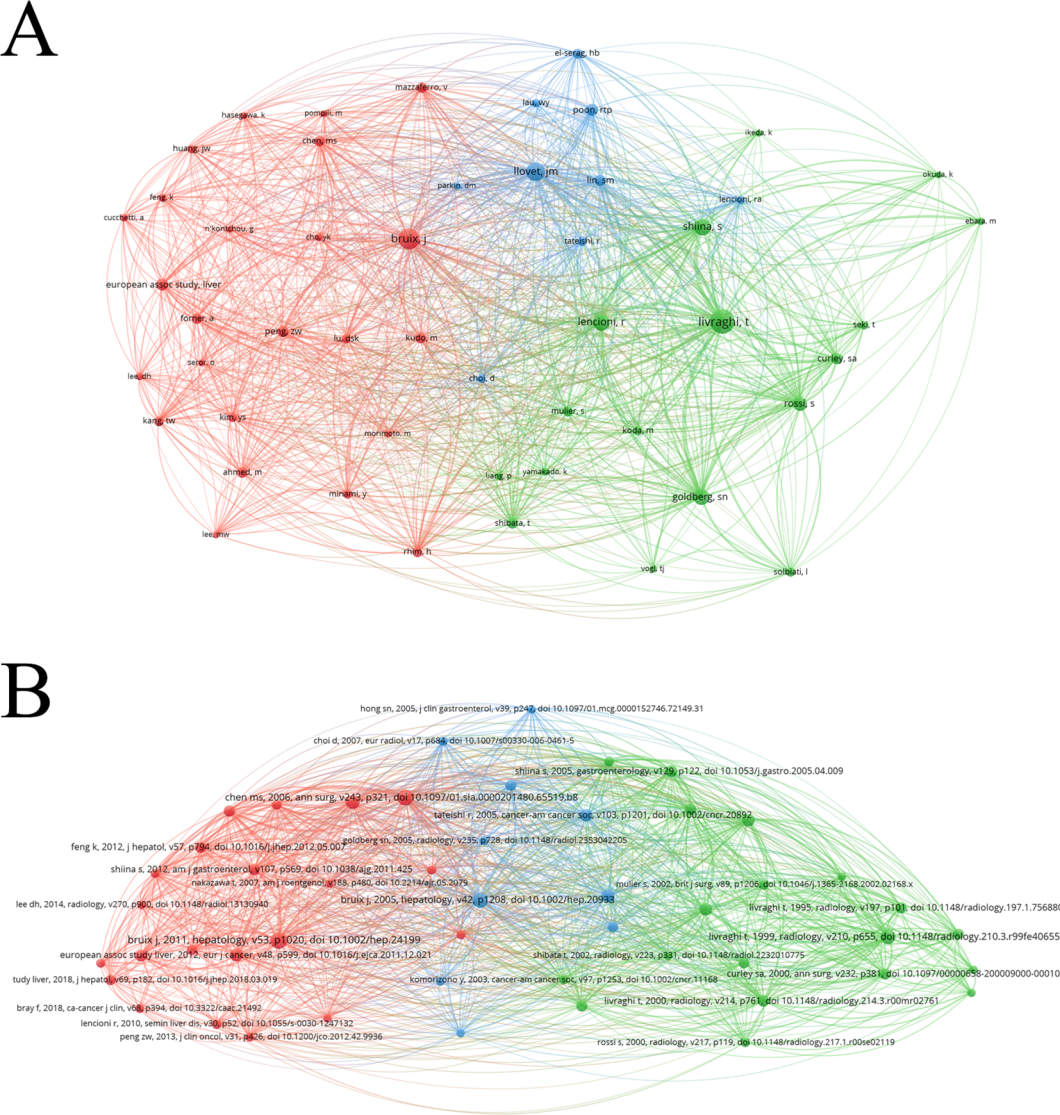
**(A)** Network visualization map of co-cited authors related to ablation treatment of hepatocellular carcinoma created by VOSviewer. **(B)** Network visualization map of co-cited references related to ablation treatment of hepatocellular carcinoma created by VOSviewer.

Between January 1, 1993 to December 31, 2022, 4,029 articles related to ablation therapy of HCC were published which have the 83,897 cited times and 29,405 references. The most cited article was published by Chen et al. in 2006, which confirmed that percutaneous local ablative therapy had the same therapeutic effect on small HCC as surgical resection did (17). In addition, highly cited studies include that published by Livraghi et al. in 1999, which indicated that the therapeutic effect of RFA was better than that of PEI (18), and that published by Livraghi et al. in 2000 and 2008, which indicated that RFA caused less trauma and had a lower complication rate and cost than surgical resection did (19, 20). The top 10 most cited articles all had more than 585 total citations (**Table 5**). The most cited reference was published by Bruix et al. in 2011, which is a practice guideline about the management of HCC (21). Articles with most cited times published by Chen MS and Livraghi T were also had more reference cited (**Table 6**). In addition, the most influential authors and references have close co citation relationship with each other (**Figures 4A, B**).

**Table 5:** The most cited publications.

Rank	Document	DOI	Citations
1	Chen MS, 2006, Annals of Surgery	10.1097/01.sla.0000201480.65519.b8	1059
2	Livraghi T, 1999, Radiology	10.1148/radiology.210.3.r99fe40655	1019
3	Livraghi T, 2000, Radiology	10.1148/radiology.214.3.r00mr02761	835
4	Livraghi T, 2008, Hepatology	10.1002/hep.21933	815
5	Livraghi T, 1995, Radiology	10.1148/radiology.197.1.7568806	795
6	Lencioni RA, 2003, Radiology	10.1148/radiol.2281020718	756
7	Shiina S, 2005, Gastroenterology	10.1053/j.gastro.2005.04.009	658
8	Lencioni R, 2005, Radiology	10.1148/radiol.2343040350	645
9	Tateishi R, 2005, Cancer	10.1002/cncr.20892	632
10	Bruix J, 2015, Lancet Oncology	10.1016/S1470-2045 (15)00198-9	585

**Table 6:** The most local cited references.

Rank	Document	DOI	Citations
1	Bruix J, 2011, Hepatology	10.1002/hep.24199	453
2	Chen MS, 2006, Annals of Surgery	10.1097/01.sla.0000201480.65519.b8	403
3	Bruix J, 2005, Hepatology	10.1002/hep.20933	399
4	Livraghi T, 1999, Radiology	10.1148/radiology.210.3.r99fe40655	344
5	Livraghi T, 2008, Hepatology	10.1002/hep.21933	308
6	European Assoc Study Liver, 2012, European Journal of cancer	10.1016/j.ejca.2011.12.021	286
7	Bruix J, 2001, Journal of Hepatology	10.1016/s0168-8278(01)00130-1	285
8	Tateishi R, 2005, Cancer	10.1002/cncr.20892	264
9	Lencioni RA, 2003, Radiology	10.1148/radiol.2281020718	260
10	Livraghi T, 2003, Radiology	10.1148/radiol.2262012198	256

### 3.5 Analysis of keywords

**Figure 5:**
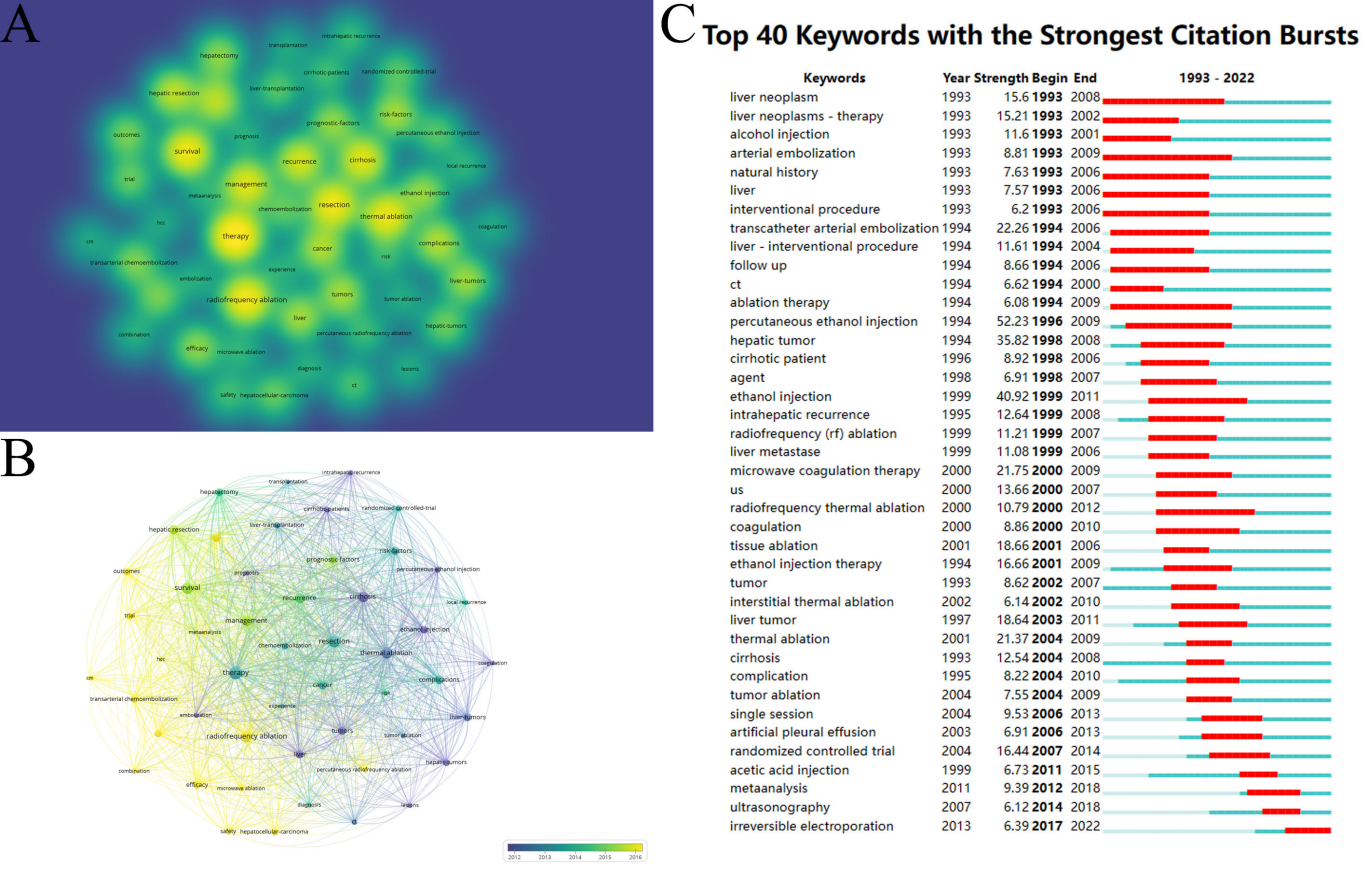
**(A)** The most common keywords in HCC ablation research. The brightness of each node represents the frequency of keywords. **(B)** Overlay visualization map of co-occurring keywords. The color of each node represents the average appearing year for the keyword, depending on the color gradient shown at the bottom. **(C)** The top 40 keywords with the strongest citation bursts during 1993–2022 (generated by CiteSpace).

We built a keyword co-occurrence network to show the connection between the high-frequency keywords for HCC ablation therapy. **Figure 5A** shows high-frequency keywords, which mainly focus on “therapy,” “resection,” “radiofrequency ablation” and “survival”. On analyzing the trend of keywords, the ablation treatment method gradually changed from PEI to RFA and MWA and the research focus changed from “complications” and “local recurrence” to “survival”, “efficacy” and “combination” (**Figure 5B**). Burst keywords were thought to be another crucial indicator of research frontiers, and they might foretell rising trends to a degree. From 1993 to 2022, **Figure 5C** shows the top 40 terms with the most citation bursts. the most recent burst keywords were “irreversible electroporation” (2017–2022), “ultrasonography” (2014 – 2018), “meta-analysis” (2012 – 2018) and “acetic acid injection” (2011 – 2015). IRE may become the main method of ablation therapy in the future.

## 4 Discussion

In this study, we systematically searched for and collected articles published in the field of HCC ablation from January 1, 1993 to December 31, 2022. After excluding studies that did not meet the screening criteria, 4,029 articles published in 464 journals from 57 countries and 2,216 institutions were analyzed. The results showed that the number of publications related to HCC ablation was on the rise every year, and the number of references was also increasing. In the 1980s, ablation of HCC was rarely performed, and the main method used was PEI (22). Later, RFA was proven to have the same therapeutic effect on small HCC as surgical resection did, with less trauma, and it gradually became the most commonly used method for HCC ablation (17, 19). At present, MWA and cryoablation have gradually become important HCC ablation methods (23, 24). Analysis of countries/regions indicated that China had the largest number of publications in the field of HCC ablation (1,281 publications), and Sun Yat-sen University had the largest number of publications in this field. In addition, Japan, Italy, the United States and Korea also attached great importance to research on HCC ablation, and they have close cooperation with each other. The top 10 journals published 1,351 articles related to HCC ablation, accounting for 33.53% of the total number. Among them, *Hepatology*, *Radiology*, *Journal of Hepatology*, and *American Journal of Roentgenology* not only published a large number of articles but also had a high impact. In addition, journals with great influence in the field of HCC ablation have a close co-citation relationship, which indicates that research on HCC ablation is professional and cooperative. Our data will help researchers select appropriate journals when submitting manuscripts related to HCC ablation therapy. In medical literature, the number of times an article is cited by other authors usually reflects the influence of the article in its field (25, 26). Among the top 10 most cited articles, 4 were published by Livraghi et al, suggesting that this author has great influence in the field of HCC ablation. The first article by Livraghi et al. was published in *Radiology* in 1995, which confirmed the safety and effectiveness of PEI for HCC (27). Subsequently, Livraghi et al. published two articles in *Radiology* in 1999 and 2000, respectively, which confirmed that RFA is superior to PEI in the treatment of HCC and is effective for medium (3.1-5.0 cm) and large (5.1-9.5 cm) HCCs (18, 20). Livraghi et al. published another article in *Hepatology* in 2008, discussing the advantages of ablation versus surgical resection in the treatment of T1 HCC (19). The most frequently cited article was published in *Annals of Surgery* by Chen et al. in 2006, which also confirmed that the therapeutic effect of local ablation on small HCC is equivalent to that of surgical resection (17). Lencioni et al. conducted a short term follow-up study, which confirmed that the RFA is superior to PEI with respect to local recurrence-free survival rates. This conclusion was published in the *Radiology* in 2003 (28). Research on keywords can indicate the research hotspots and frontiers of HCC ablation. Our research showed that the hot keywords for HCC ablation research were “therapy,” “resection,” “radiofrequency ablation” and “survival”, which indicated that the HCC ablation research mainly focused on the comparison with resection treatment and the long-term treatment effect. According to the time trend of keywords, we found that the most popular method of ablation was PEI, which later became RFA and MWA. Compared with RFA, MWA has the inherent advantages of fast ablation speed and high temperature, and it is not easily affected by heat sinks; therefore, it has been widely used worldwide in recent years (29, 30). Irreversible electroporation, a novel, nonthermal form of tumor ablation, relies on short pulses of high-frequency energy to induce pores in the lipid bilayer of cells, leading to cell death via apoptosis (31). IRE is not affected by heat sink and may result in less collateral damage based on its mechanism of action (32). Burst keywords analysis indicated IRE may become the main method of ablation therapy in the future.

At present, most HCC management guidelines regard ablation as one of the main methods for treating HCC. In the current American Association of the Study of Liver Disease guideline, locoregional treatment can be considered for patients with cirrhosis and HCC (e.g., T2 or T3 and no vascular involvement), if they are not candidates for transplantation or resection (1); the Asia-Pacific Association of the Study of Liver guideline recommends ablation as an alternative to resection for Child–Pugh class A or B patients with ≤3 tumors that are each ≤3 cm in size (33). The European Association for the Study of the Liver guideline recommends ablation as the preferred treatment for unresectable BCLC-0 and A tumors, an option for single tumors that are 2-3 cm in size, and a potential first-line therapy for resectable BCLC-0 HCC with favorable locations (34). At present, ablation combined with TACE can be used to treat patients with unresectable HCC (3–7 cm) (35). However, increasing evidence shows that ablation can not only eliminate local lesions but also play a role in remote lesions through the immune response (36). Research has shown that PEI, RFA, and MWA can activate the immune response, but these responses are relatively weak and cannot completely control the tumor (37–40). Immunotherapy is currently popular for the treatment of HCC, and nivolumab (anti PD-1), atezolizumab (anti PD-L1), and tremelimumab (anti CTLA-4) have been proven to be effective in the treatment of HCC and are widely used in clinical practice (36). Many combined applications and clinical studies of ablation and immunotherapy are currently underway (9, 41, 42). This finding suggests that ablation plays an important role in the systematic and combined treatment of HCC.

In recent years, several bibliometric studies have been conducted. The specific steps of bibliometric research can be divided into four parts: 1. define the purpose and scope of bibliometric research, 2. select the techniques that can be used for bibliometric analysis, 3. collect data for bibliometric analysis, and 4. run bibliometric analysis and report the research results. By analyzing the internal links between scientific knowledge, bibliometric research can help researchers understand knowledge trends in specific fields and become an important reference for researchers to carry out studies. However, this study had the following limitations. 1. The data retrieved in this study were all from the WOS database. Owing to the limitation of retrieval conditions, we could not include all publications in the research field. 2. Owing to language restrictions, Chinese publications were not included, which may have led to an underestimation of the contribution of Chinese researchers. 3. Owing to the continuous development of ablation technology, some new items have not been retrieved, which may have led to the omission of new research directions.

## 5 Conclusion

To our knowledge, this is the first bibliometric analysis of HCC ablation. From 1993 to 2022, the number of publications on HCC ablation has gradually increased. China is the country with the most contributions to this field, and Sun Yat-sen University is the institution with the most contributions. *Hepatology* is the journal with the most publications while *Radiology* is the most influential journal. The research direction of ablation treatment of HCC mainly focuses on “therapy,” “resection,” “radiofrequency ablation” and “survival”. The ablation treatment method has gradually changed from PEI to RFA and MWA and IRE may become the main method of ablation therapy in the future. The research focus changed from “complications” and “local recurrence” to “survival”, “efficacy” and “combination”. This analysis provides an accessible list for surgeons, oncologists, and hepatobiliary practitioners, reveals the development process of HCC ablation, and determines potential future research directions.

## Data availability statement

The original contributions presented in the study are included in the article/supplementary material. Further inquiries can be directed to the corresponding author.

## Author contributions

All authors listed have made a substantial, direct, and intellectual contribution to the work, and approved it for publication.
